# LGALS3BP is a novel and potential biomarker in clear cell renal cell carcinoma

**DOI:** 10.18632/aging.205578

**Published:** 2024-02-22

**Authors:** Lei Li, Sen Qin, Hongwei Tan, Jiexue Zhou

**Affiliations:** 1 Department of Urology, Union Hospital, Tongji Medical College, Huazhong University of Science and Technology, Wuhan, Hubei, People’s Republic of China; 2 Department of Orthopedics, The First People’s Hospital of Jingzhou, Jingzhou, Hubei, People’s Republic of China; 3 Department of Organ Transplantation, Guangdong Second Provincial General Hospital, Guangzhou, Guangdong, People’s Republic of China

**Keywords:** bioinformatics, biomarkers, LGALS3BP, clear cell renal cell carcinoma, drug screening

## Abstract

Clear cell renal cell carcinoma (ccRCC) is the most common solid renal tumor. Therefore, it is necessary to explore the related tumor markers. LGALS3BP (galectin 3 binding protein) is a multifunctional glycoprotein implicated in immunity and cancer. Some studies have shown that LGALS3BP promotes the occurrence and development of tumors. However, their exact role in renal tumorigenesis remains unclear. Our study used a webserver to explore the mRNA expression and clinical features of LGALS3BP in ccRCC. Survival analysis showed that patients with high LGALS3BP expression had significantly worse OS and DFS than those with low LGALS3BP expression. LGALS3BP expression is significantly related to B cells, CD4+ T cells, macrophages, neutrophils, and dendritic cells. Furthermore, we determined that LGALS3BP is significantly associated with angiogenesis, stemness and proliferation in renal cancer. Three phenotypes may be associated with a poor prognosis. Genes related to proliferation, angiogenesis and stemness were derived from a Venn diagram of FGF2. FGF2 is negatively correlated with proliferation and positively correlated with angiogenesis. Finally, we screened for drugs that may have potential therapeutic value for ccRCC. The PCR results showed that the expression of LGALS3BP in the normal cell line was lower than that in the tumor cell lines. After LGALS3BP knockdown, the proliferation of 769-P and 786-O cells decreased. The present findings show that LGALS3BP is critical for ccRCC cell proliferation and may be a potential target and biomarker for ccRCC.

## INTRODUCTION

Renal clear cell carcinoma (ccRCC) is the most common subtype, with the worst degree of malignancy and prognosis. According to relevant statistics, in 2021, the number of kidney cancer-related deaths in China reached 77,410 [1]. Currently, the main treatments for ccRCC include surgery, radiotherapy, and chemotherapy. However, high recurrence and metastasis rates still affect patient outcomes [2, 3]. Recent studies have shown that tumor immune infiltration plays a crucial role in the occurrence and development of tumors [4]. Therefore, exploring the immune microenvironment of ccRCC can help to identify reliable biomarkers and help clinicians treat ccRCC more accurately and effectively. LGALS3BP is a secreted multifunctional glycoprotein closely related to cancer [5], and changes in cellular pathways may be achieved through alterations in protein glycosylation, such as proliferation signaling, cell death resistance, growth inhibition evasion, angiogenesis, genome instability and mutation, invasion and metastasis, immune evasion and protumor inflammation [6, 7]. Accumulating evidence suggests that LGALS3BP plays a key role in tumor progression and metastasis. Elevated LGALS3BP expression is closely related to poor prognosis in various tumors [8–19]. Current studies have demonstrated that LGALS3BP may be a therapeutic target and biological fabric for breast cancer. LGALS3BP may be glycosylated by GALNT6 to promote autocrine cell growth; thus, the proliferation of breast cancer cells strongly correlates with the GALNT6-LGALS3BP axis [20]. LGALS3BP is significantly upregulated in OSCC tumor tissues, and the PI3K/AKT pathway is regulated by LGALS3BP and affects OSCC proliferation and migration [21]. In lung cancer, Sun et al. suggested that LGALS3BP is a potential prognostic marker [22]. Related reports have shown that LGALS3BP is also associated with drug resistance. 17-AAG resistance is mediated through PI3K/Akt activation by LGALS3BP [23]. There are significant differences in the expression of LGALS3BP in prostate cancer, and some research reports have shown that high LGALS3BP promotes the occurrence, proliferation, differentiation and metastasis of cancer cells [24]. Our study is the first to investigate the expression of LGALS3BP in ccRCC and its relationship with clinical features and prognosis. We combined clinically relevant factors and LGALS3BP expression to construct a predictive nomogram for clinical prediction. We used various network tools to study the immune infiltration, functional status, and potential biological functions of LGALS3BP in ccRCC progression, which will help in understanding the possible mechanisms of renal carcinogenesis. Additionally, we performed LGALS3BP expression detection and cell function experiments to validate our results. Finally, we screened drugs that may have potential therapeutic value for ccRCC. It is hoped to provide new biomarkers for ccRCC and would lead to a better understanding of the possible role of LGALS3BP and its prognostic value in ccRCC.

## RESULTS

### Expression patterns of LGALS3BP

**Figure 1:**
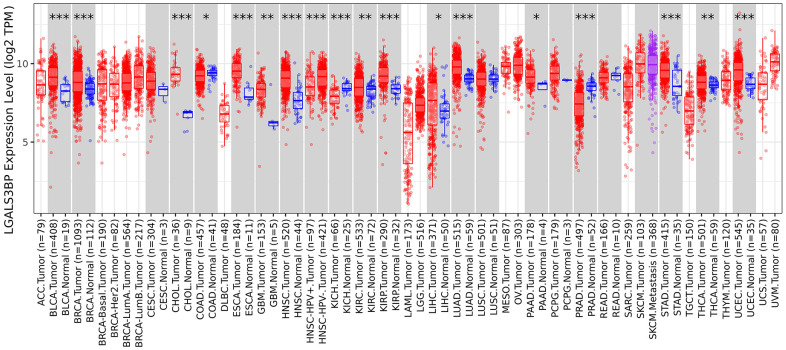
Expression level of LGALS3BP in pancancer.

**Figure 2:**
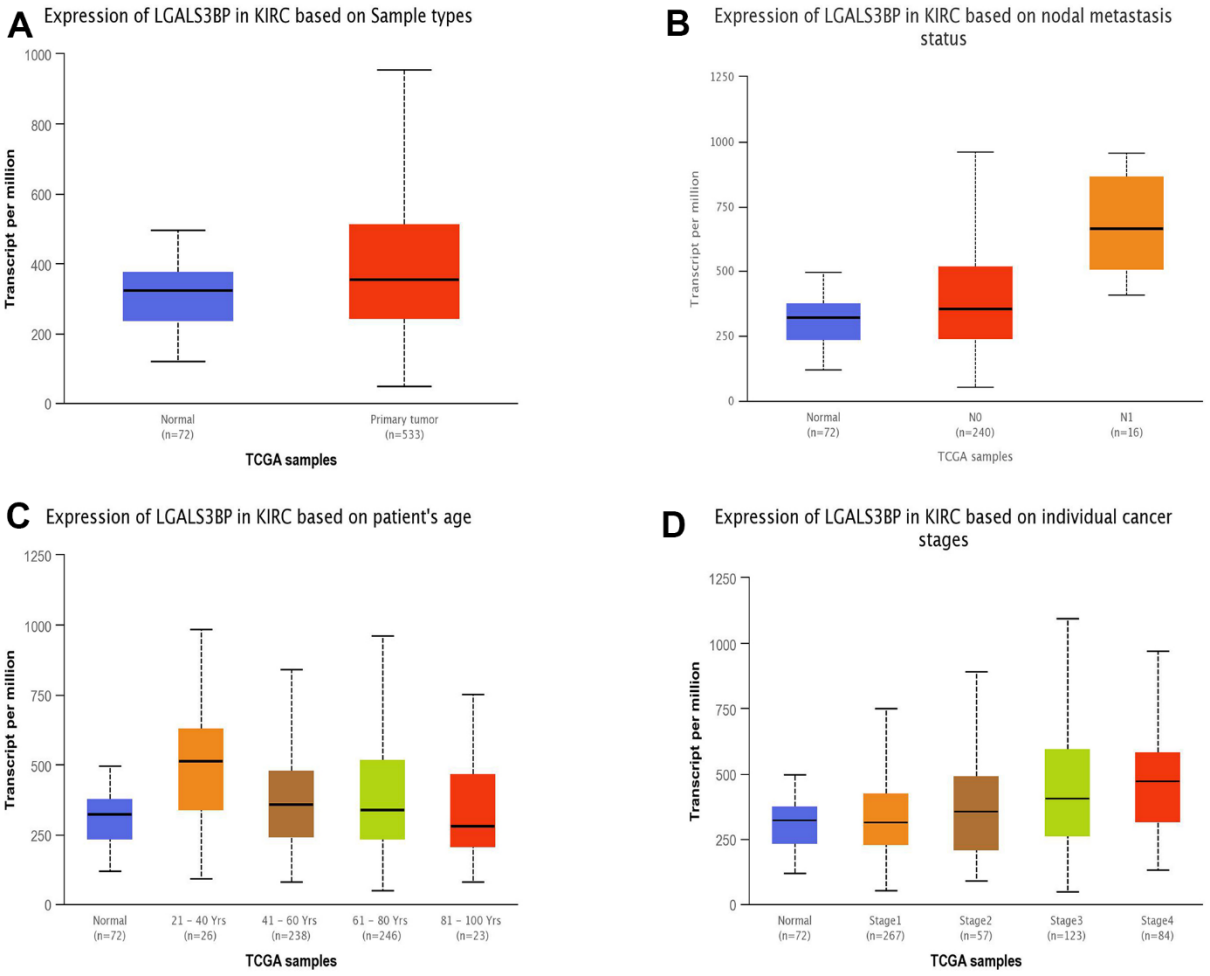
**The expression of LGALS3BP mRNA in subgroups of ccRCC patients (UALCAN).** (**A**) The comparative expression of LGALS3BP in normal and KIRC samples. (**B**) LGALS3BP expression in normal and ccRCC tissues (with or without nodal metastasis). (**C**) LGALS3BP expression in ccRCC patients aged 21–40, 41–60, 61–80, or 81–100. (**D**) Comparative expression of LGALS3BP in normal and ccRCC (stage 1, 2, 3, or 4) samples. *, p < 0.05; **, p < 0.01; ***, p < 0.001.

The TIMER 2.0 results showed obvious differences in the expression of LGALS3BP in pancancer (Figure 1) (Supplementary Table 1). In the present study, LGALS3BP was high expressed in tumors compared to normal tissue. LGALS3BP correlated with clinical features, such as metastasis, age, and stage (Figure 2A–2D). The results showed that the transcriptome level of LGALS3BP in the normal group was lower than that in the tumor group.

### Correlation between the expression of LGALS3BP and clinicopathological features

**Figure 3:**
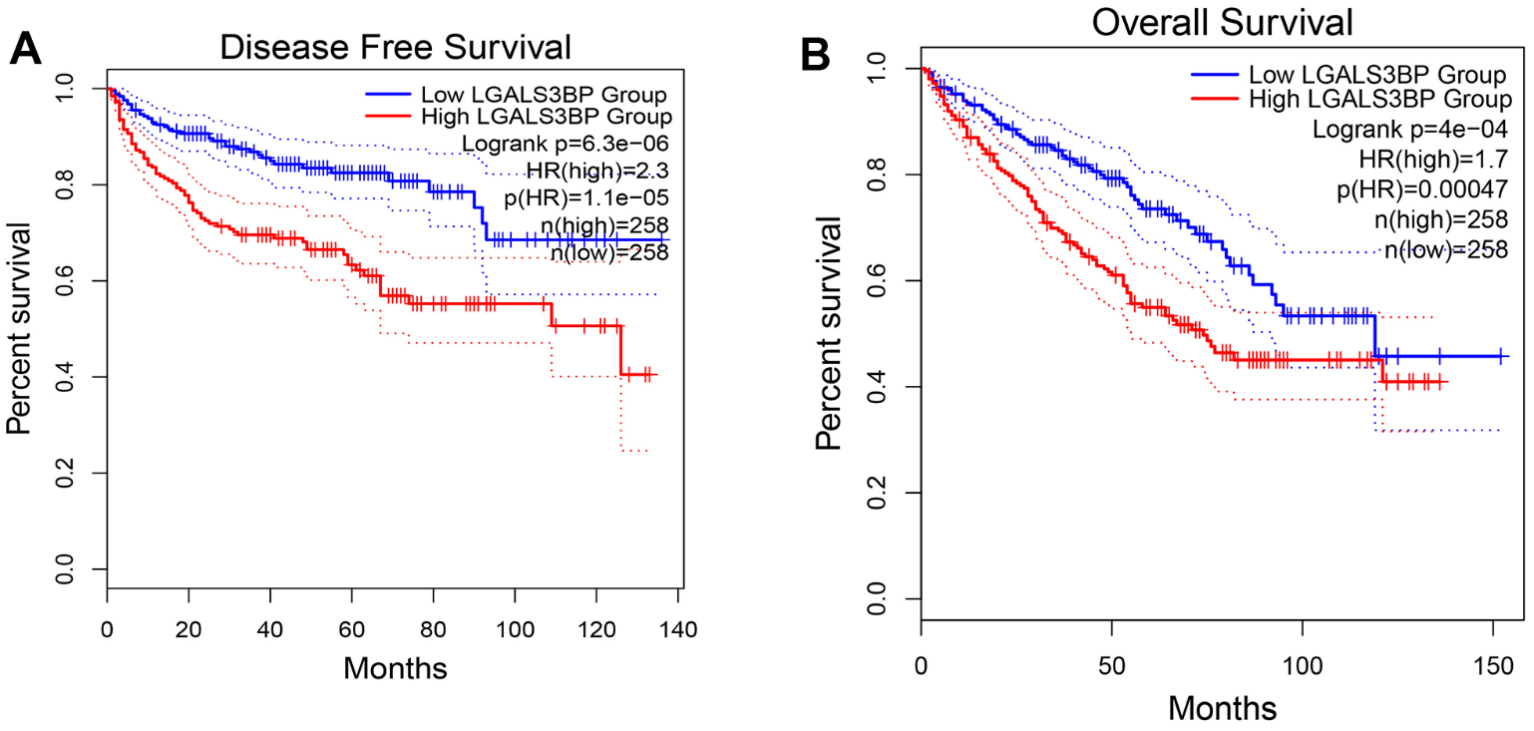
**The prognostic value of LGALS3BP in KIRC.** (**A**) Disease-free survival in ccRCC patients with high or low LGALS3BP expression (GEPIA2). (**B**) Overall survival in ccRCC patients with high or low expression of LGALS3BP (GEPIA2).

**Figure 4:**
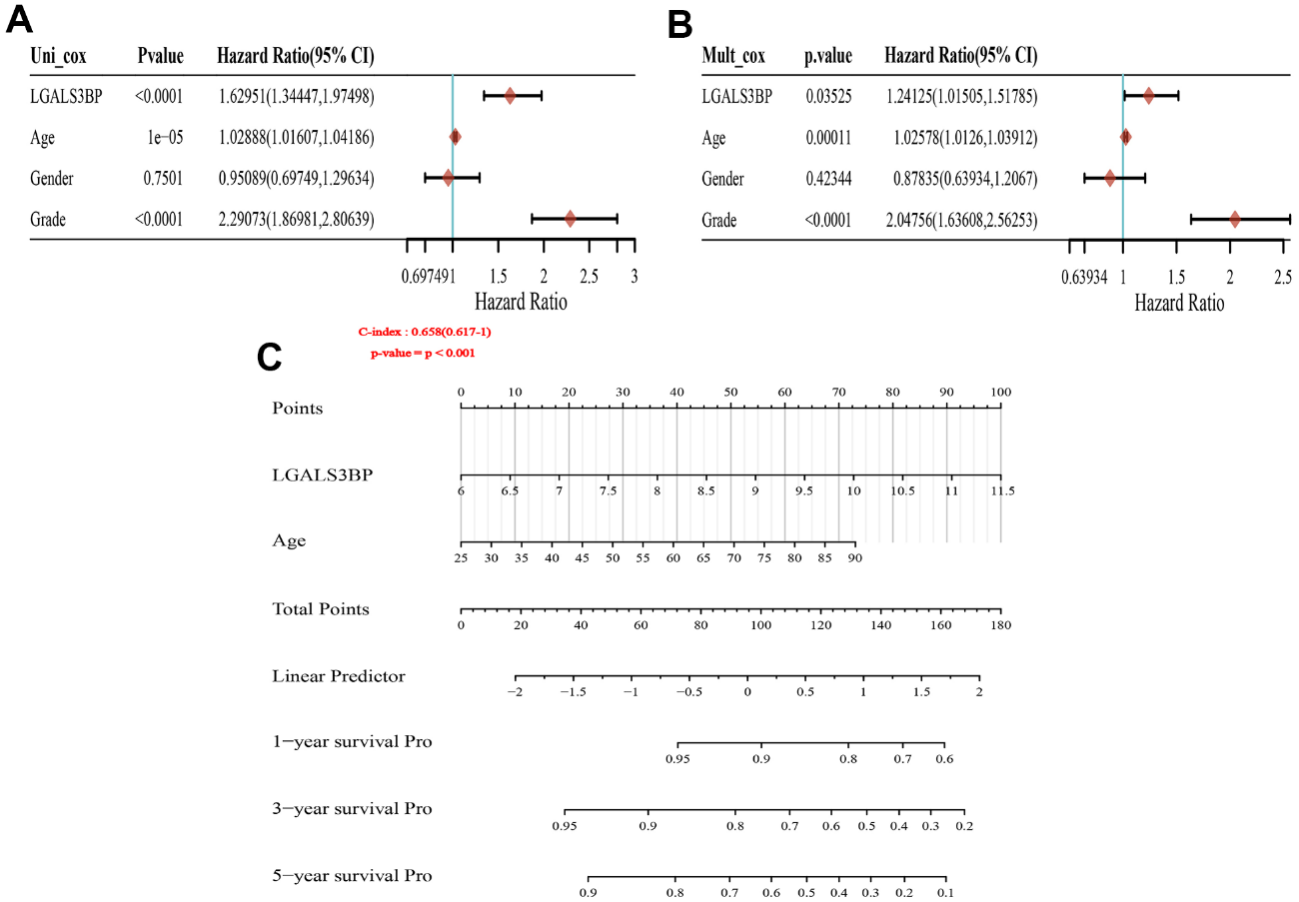
**Forest plot and nomogram construction.** (**A**) Forest plot of the association between risk factors and overall survival (OS) in TCGA-KIRC patients. (**B**) Prediction model of nomogram construction. (**C**) Constructing the clinicopathological characteristics model of the Norman diagram. p < 0.001.

The GEPIA2 results showed that OS and DFS were positively correlated with LGALS3BP (Figure 3A, 3B). High LGALS3BP expression predicts poor prognosis. To identify independent prognostic factors in patients with ccRCC, the results of univariate analysis revealed that prognosis was related to age, grade, and LGALS3BP expression (Figure 4A). Multivariate analysis revealed that LGALS3BP was an independent prognostic risk factor for ccRCC (Figure 4B). The above analysis indicated that LGALS3BP expression significantly affected the OS and PFS of patients with ccRCC. The construction of Norman diagrams was helpful for clinical applications (Figure 4C).

### Effects of LGALS3BP on immune cell infiltration

**Figure 5:**
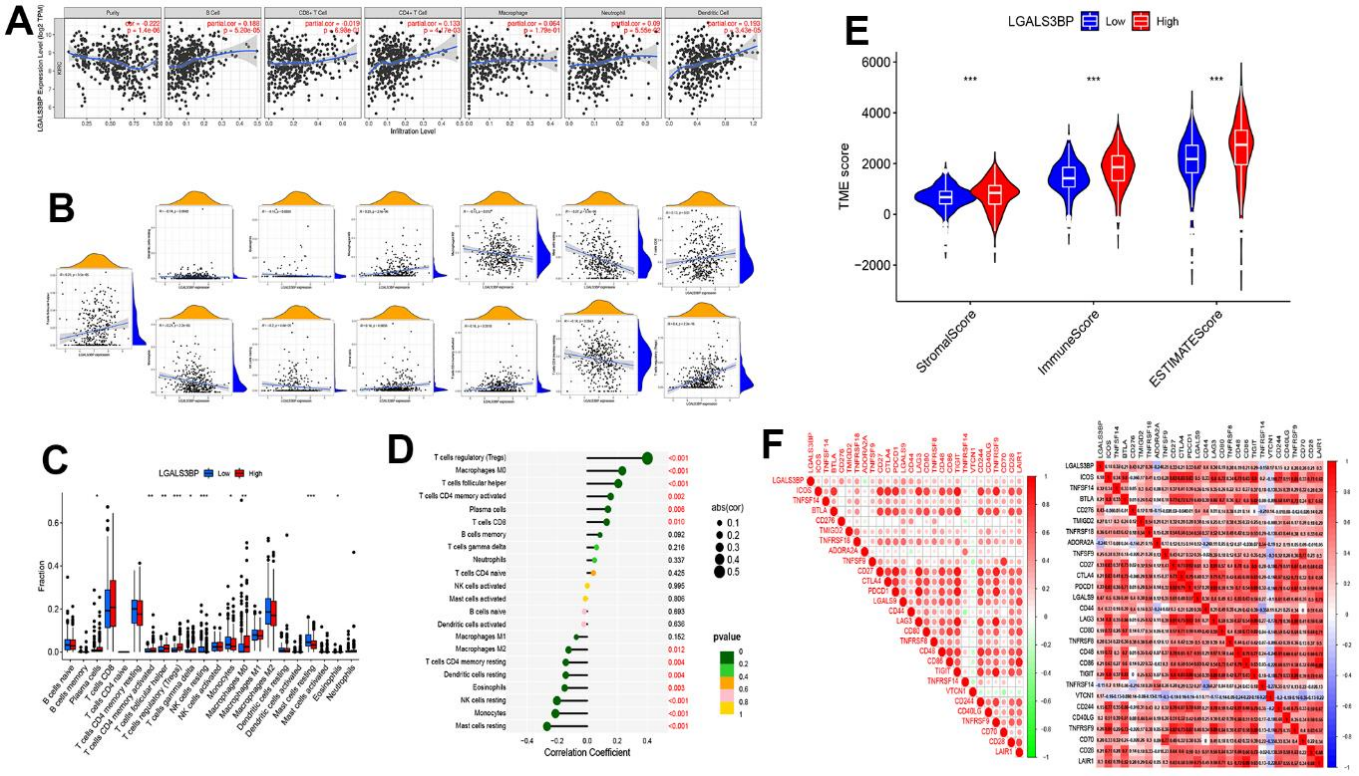
**The expression of LGALS3BP was shown to be significantly linked to immune cell infiltration.** (**A**–**D**) The relationship between immune cell infiltration and LGALS3BP. (**E**) The relationship between the TME score and LGALS3BP. (**F**) The relationship between the checkpoint and LGALS3BP.

The TIMER results proved that LGALS3BP was significantly related to renal clear cell immune cells. B cells, CD4^+^ T cells, and dendritic cells positively correlated with the expression of LGALS3BP (Figure 5A). In addition, we included more immune cells for the analysis based on LGALS3BP expression.

M0 macrophages, plasma cells, activated memory CD4 T cells, CD8 T cells, follicular helper T cells, and regulatory T cells (Tregs) were positively correlated with the expression of LGALS3BP. M2 macrophages, resting mast cells, monocytes, resting NK cells, and resting memory CD4 T cells were negatively correlated with the expression of LGALS3BP (Figure 5B). The results of immune infiltration are displayed in the graph (Figure 5C, 5D). The tumor microenvironment score was significantly different between the high and low groups (Figure 5E). High LGALS3BP expression indicated rich immune infiltration. Therefore, we next explored whether there were any differences in immune checkpoints between the two groups. The correlative map results showed that the expression of ICOS, TNFSF14, BTLA, CD276, TMIGD2, TNFRSF18, TNFSF9, CD27, CTLA4, PDCD1, LGALS9, CD44, LAG3, CD80, TNFRSF8, CD48, CD86, TIGIT, VTCN1, CD244, CD40LG, TNFRSF9, CD70, CD28, and LAIR1 was significantly positively correlated with LGALS3BP. ADORA2A and TNFRSF14 were negatively correlated with LGALS3BP (Figure 5F).

### Functional status of LGALS3BP in renal cancer types

**Figure 6:**
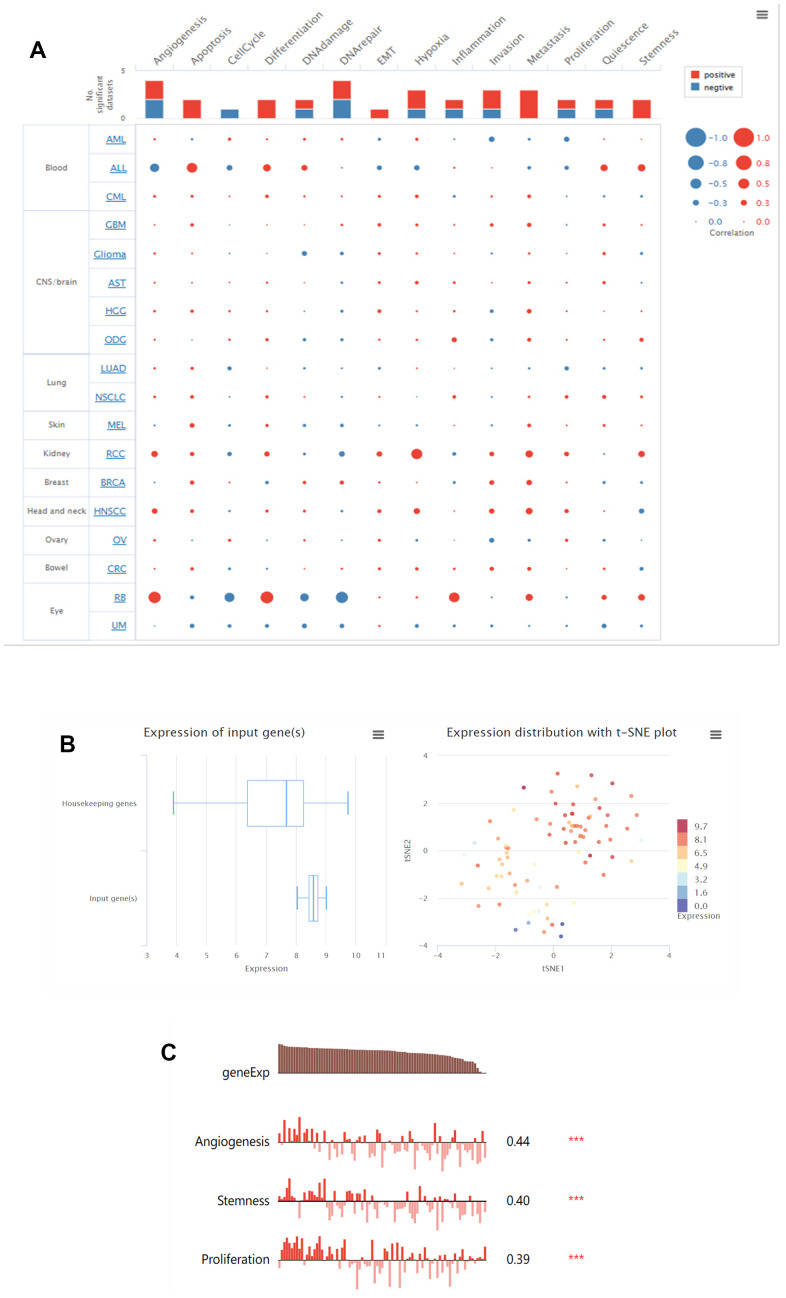
**LGALS3BP functional states in the scRNA-seq datasets.** (**A**) Relevance of LGALS3BP across 14 functional states in distinct cancers. (**B**) The overall expression of the module in ccRCC was found to be heterogeneous. (**C**) Correlations between the LGALS3BP expression and two functional states were identified by the CancerSEA database. * p < 0.05, **, p < 0.01, *** p < 0.001.

**Figure 7:**
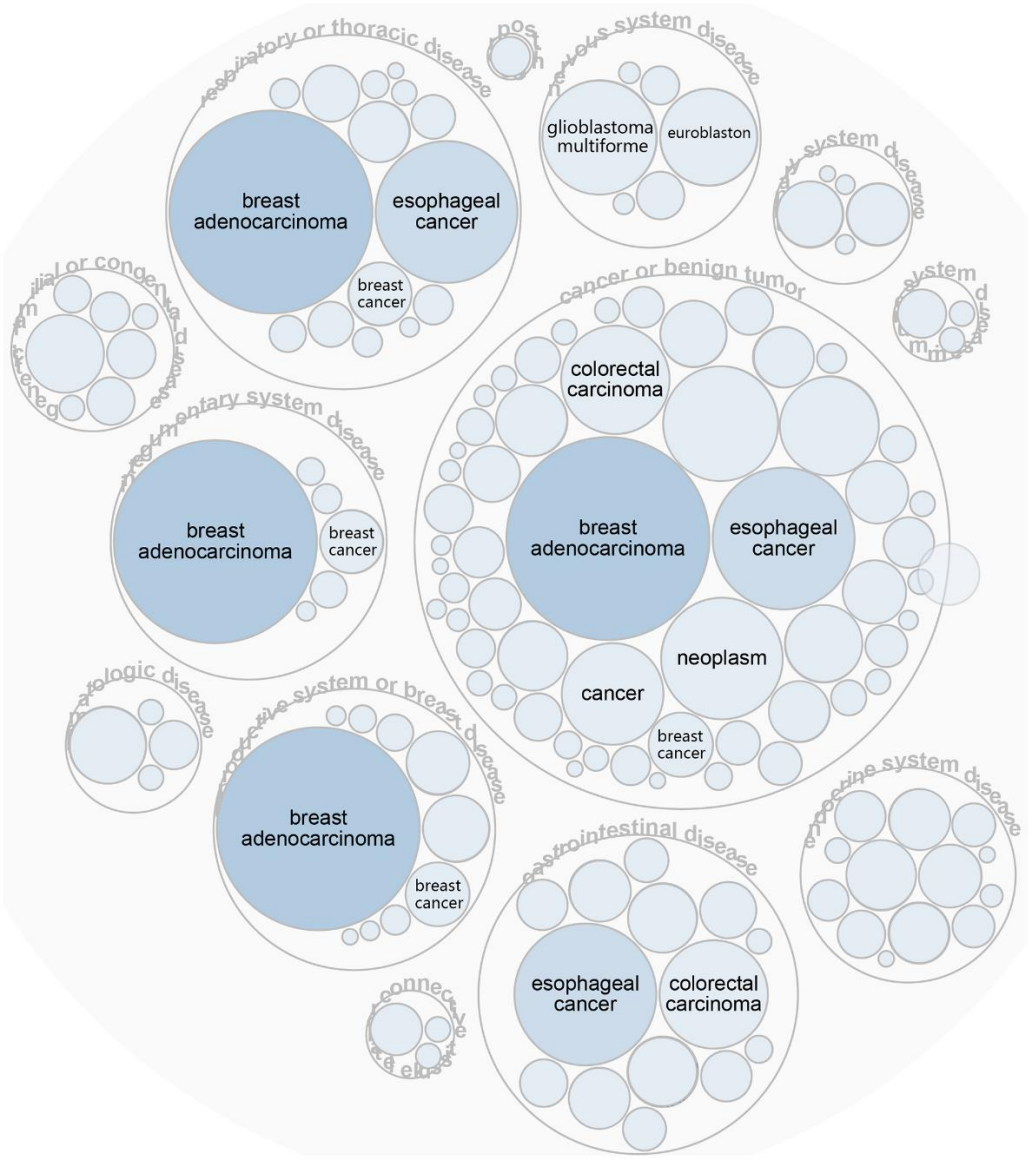
LGALS3BP has been implicated in immune and urological diseases.

In the CancerSEA database, the functional status of LGALS3BP has been analyzed in pancancer. Using a single-cell dataset, the expression of LGALS3BP and the behavior of each functional state were displayed through an interactive bubble plot. The correlation between LGALS3BP and various tumor functional states showed that renal cancer is associated with multiple biological phenotypes (Figure 6A). The expression of LGALS3BP was higher than that of the housekeeping genes and according to the clustering results of CancerSEA, the expression of LGALS3BP in RCC was heterogeneous (Figure 6B). LGALS3BP was positively correlated with angiogenesis (P<0.001, Cor=0.44), stemness (P<0.001, Cor=0.40), and proliferation (P<0.001, Cor=0.39) in ccRCC (Figure 6C). To further explore its functions, we used Open Targets to explore diseases that may be related to LGALS3BP. LGALS3BP has been implicated in immune and cancer diseases (Figure 7). These results demonstrate that LGALS3BP may be an important prognostic factor in ccRCC.

### Correlation with stemness index and clinical features

**Figure 8:**
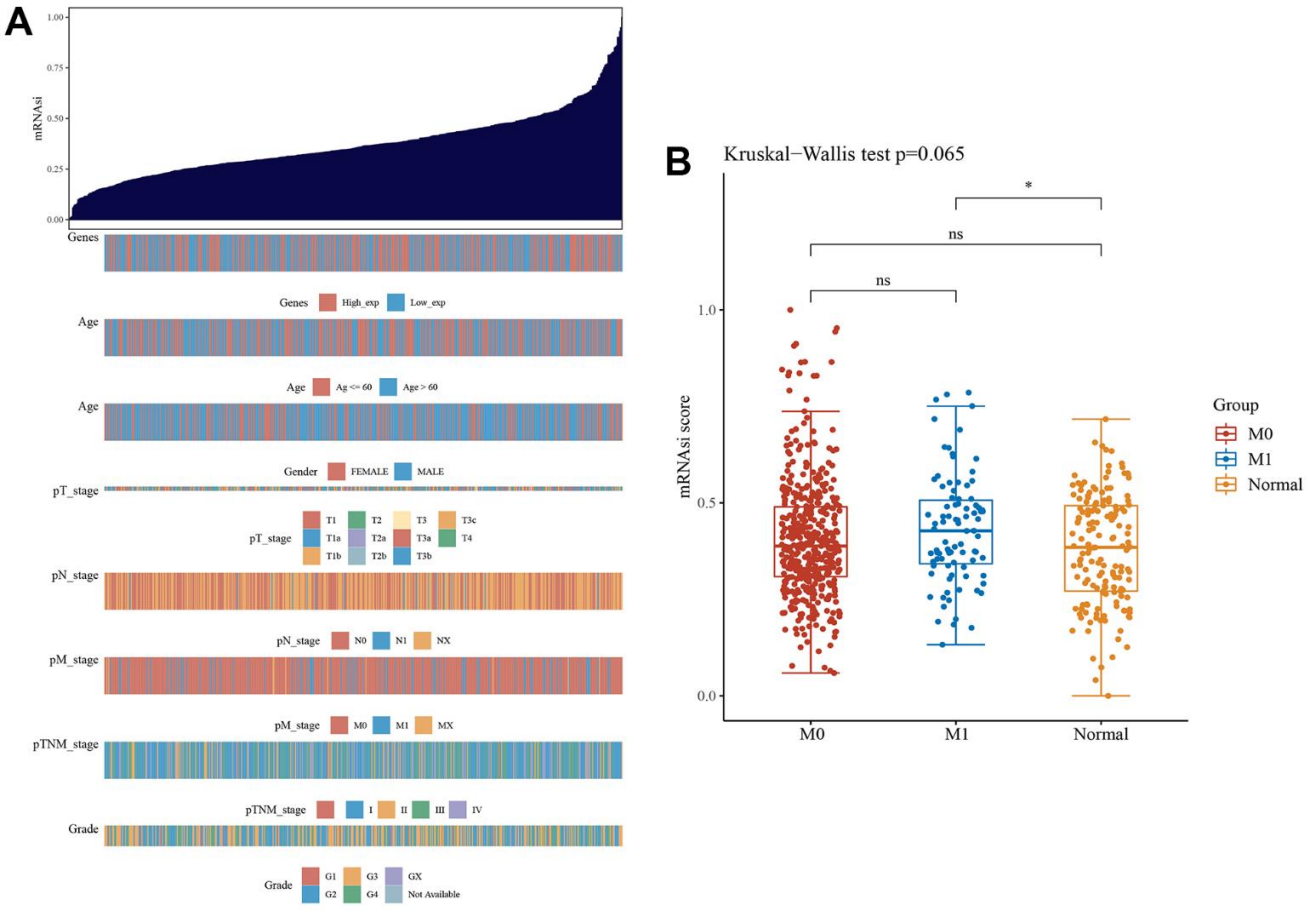
**The clinical and molecular features associated with the stemness index (mRNAsi) in ccRCC patients.** (**A**) An overview of the association between mRNAsi and clinicopathological features of patients. Columns represent samples ranked by mRNAsi from low to high (top row), and rows represent known clinical and molecular characteristics associated with mRNAsi. (**B**) The cancer stemness index of the tumor metastasis group was significantly higher than that of the normal group (p <0.05).

The results of the stemness index algorithm are displayed as a heatmap, and the relationship between the clinical features and stemness index of ccRCC was explored (Figure 8A). High LGALS3BP expression predicted a high stemness index. Similarly, the stemness index of metastatic tumors was higher than that of nonmetastatic tumors. Boxplots further demonstrate our results (Figure 8B).

### Expression of three phenotype-related genes and common related genes

**Figure 9:**
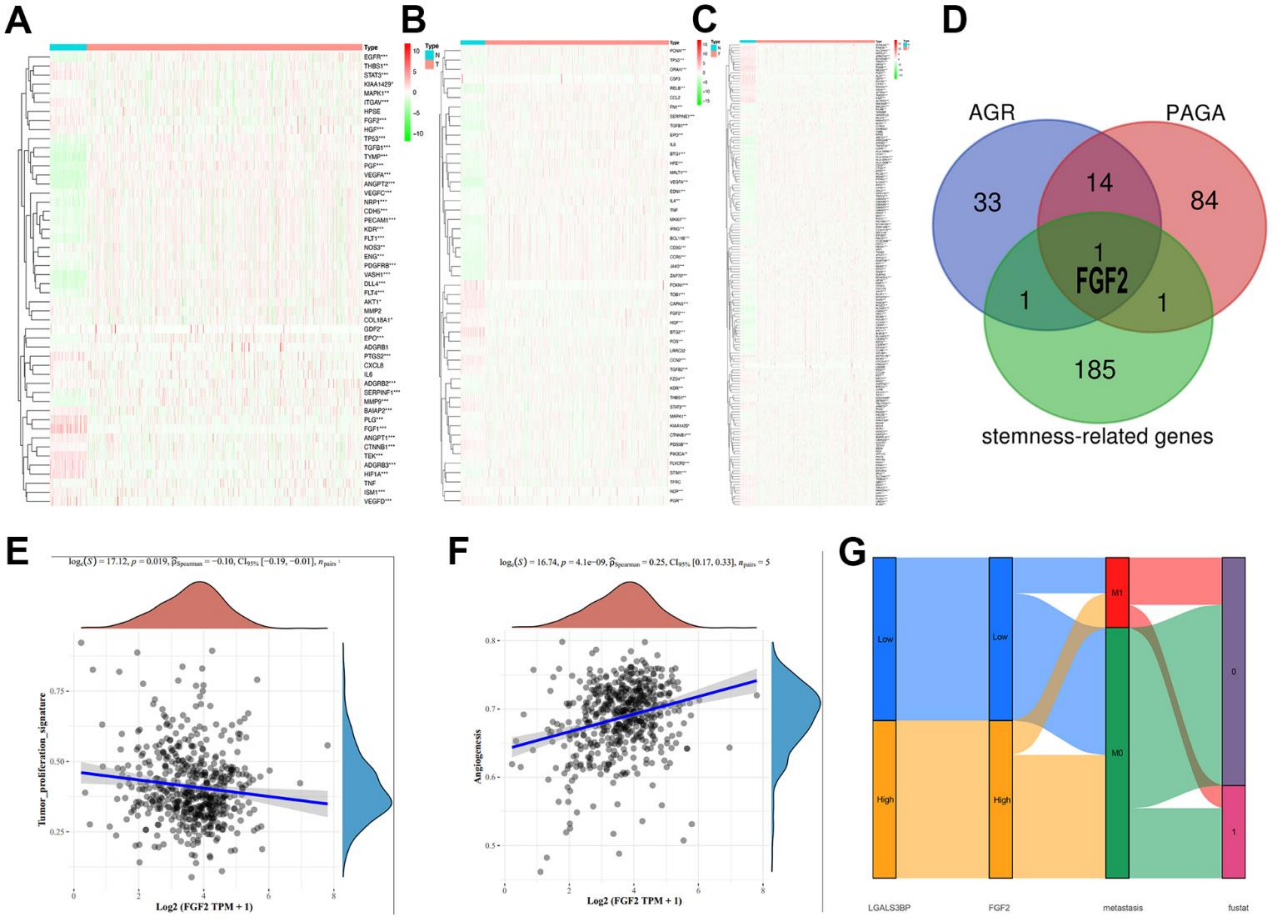
**Expression patterns of the three phenotypes in ccRCC.** (**A**) Angiogenesis-related genes. (**B**) Proliferation-related genes. (**C**) Stemness-related genes. (**D**) Cross-analysis was performed on the three groups of related genes. (**E**) Correlation of FGF2 with proliferation. (**F**) Correlation of FGF2 with angiogenesis. (**G**) The expression of LGALS3BP and FGF2, and metastatic traits associated with prognosis.

The heatmap results are shown below (Figure 9A–9C). Among the genes related to proliferation, stemness, and angiogenesis, most genes were significantly highly expressed in the tumor group. However, the specific genetic mechanism remains to be explored. Venn diagram results showed that FGF2 interacted with the three groups (Figure 9D). Unfortunately, the expression of FGF2 in the heatmaps is unclear. Therefore, we analyzed the relevance of FGF2 in proliferation and angiogenesis (Figure 9E–9F). Interestingly, FGF2 expression was negatively correlated with proliferation (P=0.019) and positively correlated with angiogenesis (P=4.1e-09). The Sankey diagram shows that LGALS3BP and FGF2 high and low expression and metastasis have obvious effects on survival (Figure 9G). FGF2 has been implicated in cancer development (Supplementary Figure 1).

### LGALS3BP expression in ccRCC

**Figure 10:**
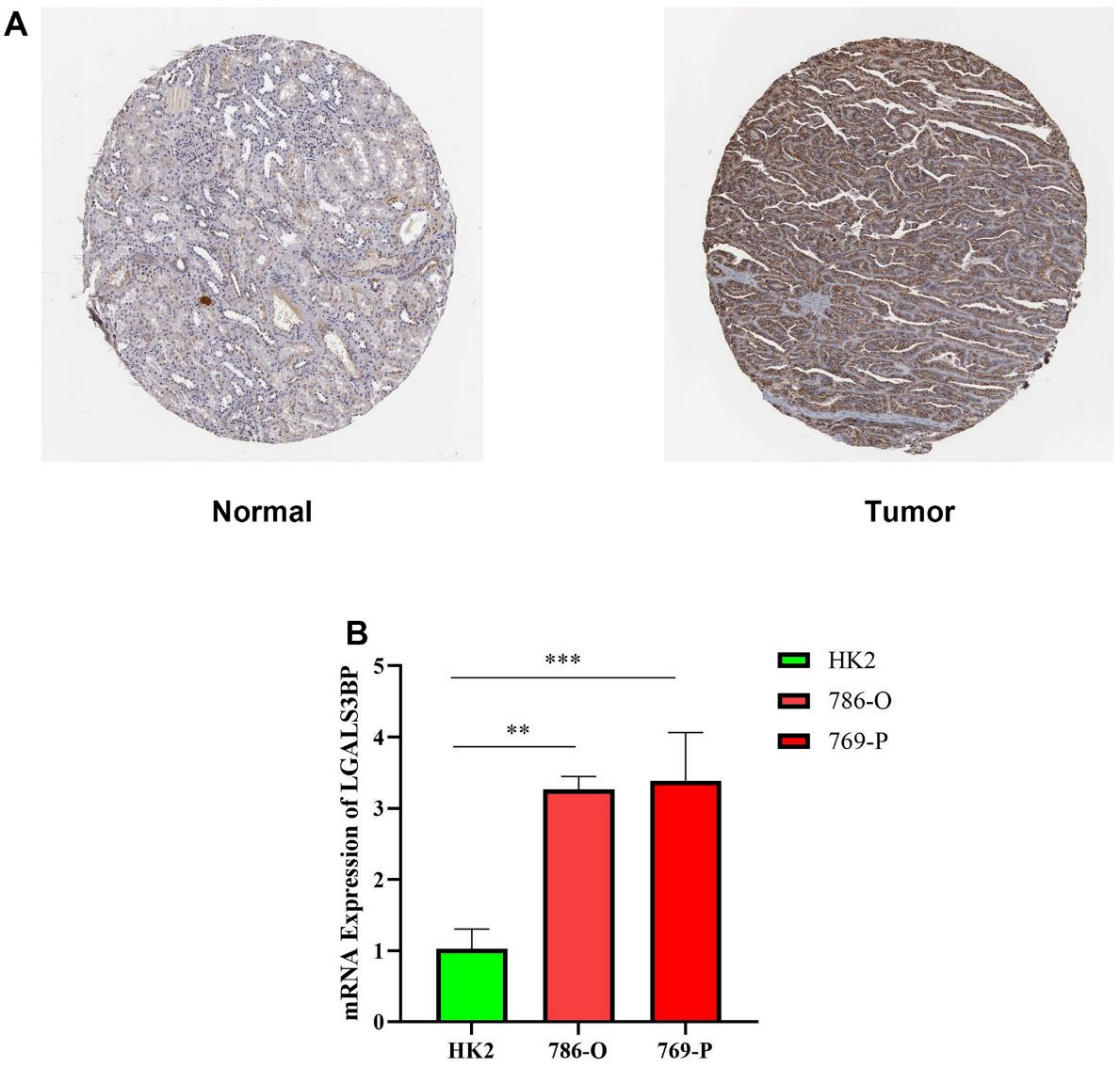
**Expression level of LGALS3BP in renal clear cell carcinoma (KIRC).** (**A**) Protein expression of LGALS3BP in KIRC. KIRC, kidney renal clear cell carcinoma. (**B**) The expression of LGALS3BP in ccRCC cell lines (HK2, 769, 786) was measured by the qRT-PCR. *p < 0.05, **p < 0.01, ***p < 0.001, and ****p < 0.0001.

We used HPA to further reveal the protein expression status of LGALS3BP in the tissues. Relative to normal kidney tissue, the level of LGALS3BP protein in RCC tissues was significantly higher (Figure 10A). HK2 (epithelial; control) and ccRCC cell lines were used to validate the mRNA expression of LGALS3BP in ccRCC. Therefore, we compared LGALS3BP mRNA levels in tumor and normal tissues to determine the LGALS3BP expression status. LGALS3BP mRNA levels in ccRCC cell lines were higher than those in the HK2 cell line (Figure 10B), consistent with our other findings.

### Proliferation of ccRCC cells can be achieved by knockdown of LGALS3BP

**Figure 11:**
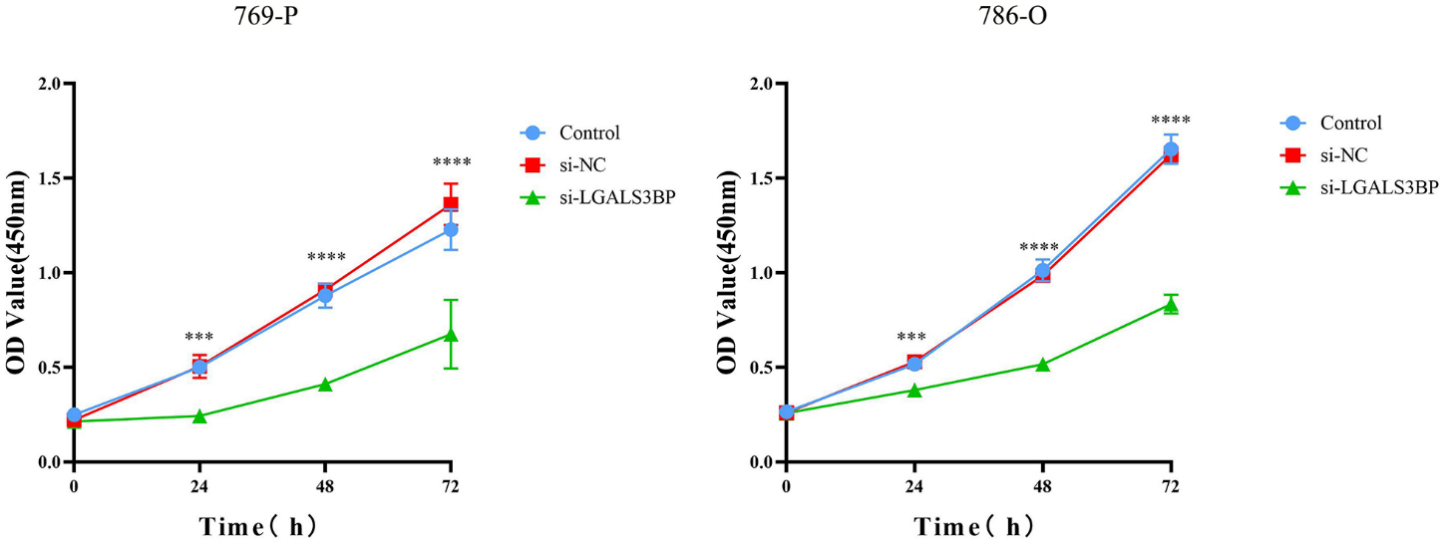
**Knockdown of LGALS3BP inhibited ccRCC cell proliferation.** A CCK-8 assay was used to detect the effect of LGALS3BP on the proliferation of 769-P cells.

To test whether LGALS3BP promoted tumor proliferation in ccRCC, we synthesized LGALS3BP-siRNA and transfected 769-P cells. We observed that LGALS3BP knockdown significantly decreased LGALS3BP mRNA expression in 769-P cells. The CCK-8 results showed that the proliferation ability of 769-P cells was significantly weakened after LGALS3BP knockdown (Figure 11).

### Screening potential drugs for ccRCC

**Figure 12:**
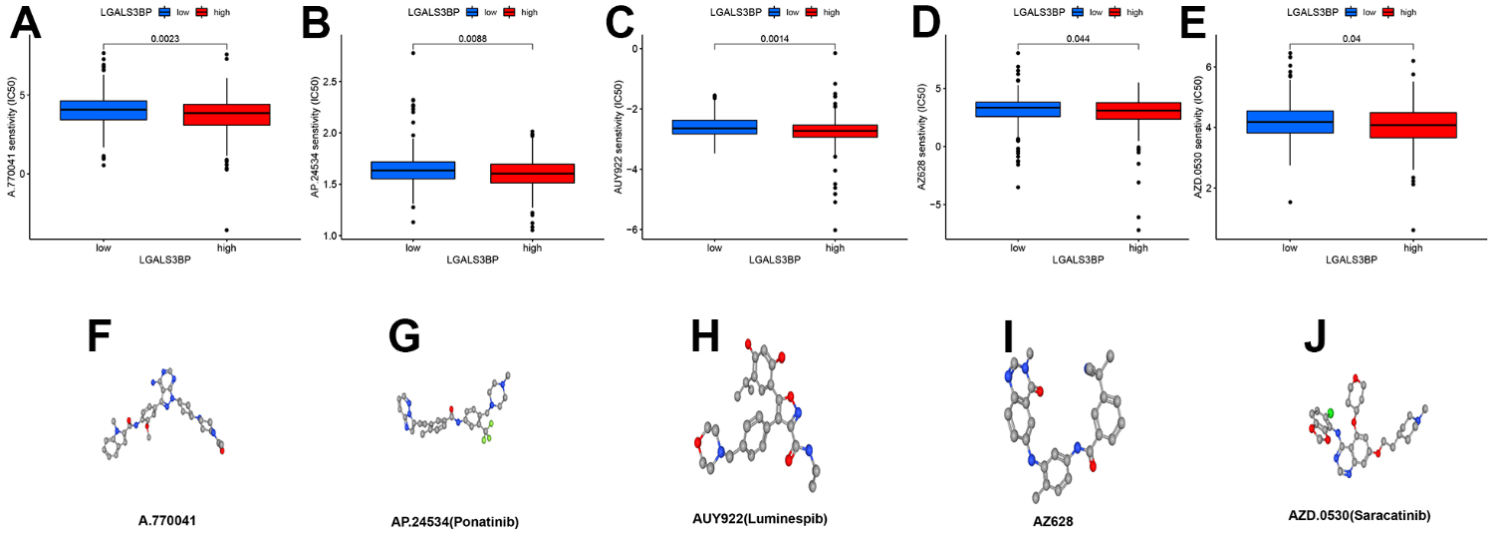
**Potential therapeutic drugs related to ccRCC and 3D conformer of five potential drugs.** (**A**) A.770041. (**B**) AP.24534. (**C**) AUY922. (**D**) AZ628. (**E**) AZD. 0530. (**F**) 3D conformer of A.770041. (**G**) 3D conformer of AP. 24534. (**H**) 3D conformer of AUY922. (**I**) 3D conformer of AZ628. (**J**) 3D conformer of AZD.0530.

Based on the median LGALS3BP level, we further divided the patients into high and low groups. The R results showed that we obtained five potential drugs for ccRCC (A.770041, AP. 24534, AUY922, AZ628, and AZD.0530) (Figure 12A–12E). The high LGALS3BP group was more sensitive to these five drugs than the low LGALS3BP group. These five drugs may play a vital role in the treatment of ccRCC and provide more drug options for the clinic. However, the specific mechanism of renal clear cell carcinoma needs to be further studied. The molecular space configuration is shown in Figure 12F–12J. P-values are less than 0.05.

## DISCUSSION

Monoclonal antibodies in human breast cancer cells can recognize LGALS3BP (a 90 kDa tumor-associated antigen) and in human lung cancer cells [5]. LGALS3BP has intracellular activity and is primarily involved in the regulation of innate immune responses [25]. Furthermore, intracellular centriole biogenesis and centrosome hypertrophy in cancer cells can be regulated by LGALS3BP to inhibit TAK1-dependent NF-κB activation [26]. Recently, numerous studies have demonstrated a significant association of LGALS3BP with tumor progression and spread, and an increasing number of studies support the drivers of different processes by which LGALS3BP leads to cellular transformation [5]. Although Minamida et al*.* reported the study of LGALS3BP in RCC, their study was limited to the protein expression level of LGALS3BP and did not explore the relationship between LGALS3BP and the cellular function of ccRCC [27]. Therefore, we will guide future ccRCC studies based on public sequencing data and use bioinformatics analyses to obtain a more comprehensive understanding of the possible functions of LGALS3BP in ccRCC. Our results showed that in the normal population, the transcript level of LGALS3BP was lower than that in ccRCC patients. The results of the correlation analysis showed that LGALS3BP was significantly associated with grade (p<0.001) and age (p<0.011). Interestingly, we detected a significantly worse prognosis with high LGALS3BP than with low LGALS3BP in ccRCC.

Multivariate Cox analysis proved that the expression of LGALS3BP was a predictor of OS in patients with ccRCC. Forest plots revealed that the clinicopathological features related to poor prognosis in ccRCC included grade, age, and LGALS3BP expression. Next, we integrated clinicopathological features and the expression of LGALS3BP to construct a nomogram survival prediction map to obtain more accurate predictions for evaluating the 1-, 3-, and 5-year survival rates of KIRC patients (C-Index is 0.658 (95% CI, 0.671-1)). The prediction model had good accuracy.

At present, there are numerous studies on the tumor immune microenvironment in various tumors. Immunotherapy brings hope to cancer patients. Immunotherapy may provide new treatment ideas to clinicians for various renal cancers with poor curative effects. TIMER analysis showed that LGALS3BP expression is related to KIRC immune infiltration. The expression of LGALS3BP correlated with various immune markers of KIRC. Our results showed that LGALS3BP was obviously correlated with B cells (Cor=0.188, p=5.20e-05), CD4+ T cells (Cor=0.133, p=4.17e-03), macrophages (Cor=0.064, p=1.79e-01), neutrophils (Cor=0.09, p=5.55e-02) and dendritic cells (Cor=0.193, p=3.43e-05). Immune checkpoint analysis showed that the expression of ICOS, TNFSF14, BTLA, CD276, TMIGD2, TNFRSF18, TNFSF9, CD27, CTLA4, PDCD1, LGALS9, CD44, LAG3, CD80, TNFRSF8, CD48, CD86, TIGIT, VTCN1, CD244, CD40LG, TNFRSF9, CD70, CD28, and LAIR1 was significantly positively correlated. ADORA2A and TNFRSF14 levels were negatively correlated with LGALS3BP. According to the previous conclusion, the high-expression group had more abundant immune infiltration. Therefore, it is reliable to administer immune checkpoint treatment for LGALS3BP. Another bioinformatics study suggested that these immune checkpoints may have prognostic value in ccRCC [28]. Further exploration of Open Targets demonstrated that LGALS3BP plays a vital role in the immune system, cancer, and urological diseases. Therefore, we speculate that LGALS3BP may have an impact on tumor immunity and may be a target for ccRCC immunity therapy. According to the CancerSEA results, LGALS3BP was positively associated with angiogenesis, proliferation and stemness in RCC. These results are consistent with previous differential expression analysis and survival analysis.

The results of the cancer stemness index proved that the index was obviously related to LGALS3BP expression and tumor metastasis. The stemness index of the metastasis group was obviously different from that of the nonmetastasis group. A study reported that predicting tumor metastasis can be achieved by the stemness index in gastrointestinal stromal tumors [29]. In addition, Wang et al*.* reported that cancer stem cells (CSCs) accelerated the development of metastatic RCC [30]. Therefore, we speculate that the stemness score may be used as a prognostic evaluation in ccRCC.

Fibroblast growth factor 2 (FGF2) is a representative paracrine FGF that binds to heparan sulfate proteoglycans and fibroblast growth factor receptor (FGFR). It has been reported that FGFR inhibitors directly exert antitumor effects on cancer cells and indirectly exert antitumor effects by blocking paracrine signaling. A study proved that FGF can suppress cancer by targeting immune evasion and angiogenesis in the tumor microenvironment [31]. Our study shows that FGF2 is inversely associated with proliferation. According to Chen et al*.*, overexpression of microRNA-936 may facilitate the occurrence of GCa, mainly by downregulating FGF2 and activating the P13K/Akt signaling pathway. Silencing FGF2 enhanced cell proliferation and invasion, which could be reversed by simultaneous downregulation of microRNA-936 [32]. Another article also reported that FGF2 has a suppressive effect on lung tumors [33]. Our results also show that FGF2 is positively correlated with angiogenesis. Huang et al*.* confirmed that the expression of FGF2 in thyroid cancer is higher than that in the normal group. In addition, FGF2 can promote angiogenesis in thyroid cancer through the regulation of the lncRNA MALAT1 [34]. FGF2 is a key regulator of melanoma angiogenesis and metastasis, and FGF2-induced melanoma angiogenesis is a heparan sulfate glycosaminoglycan chain that can be degraded by heparanase (HPSE) or HPSE (HS) regulation [35]. Finally, we combined LGALS3BP, FGF2 and metastasis to explore the prognostic impact of ccRCC patients. The results proved that compared with the nonmetastatic group, the mortality rate of patients with high expression of LGALS3BP and high expression of FGF2 in the metastatic group was much higher than that in the nonmetastatic group. FGF2 has been implicated in cancer and urological diseases. This result is highly consistent with our previous study.

In addition, by measuring the expression of LGALS3BP and the results of cell function experiments, we proved that compared with normal human renal tubular epithelial cell lines, RT-PCR and IHC results of ccRCC cell lines also proved that LGALS3BP was highly expressed in ccRCC, which is consistent with our findings. The results of previous analyses were consistent. Patients with tumors that express high levels of LGALS3BP have lower survival rates. Furthermore, the expression of LGALS3BP was inversely correlated with patient survival and was highly expressed in patients with advanced tumors and metastases. By constructing a LGALS3BP knockdown model in a ccRCC cell line, cellular phenotype testing indicated that LGALS3BP silencing could inhibit cell proliferation. Therefore, our study suggests that LGALS3BP may be an indicator of immune infiltration in ccRCC and that LGALS3BP enhances ccRCC cell proliferation and leads to poor prognosis in patients. We used LGALS3BP as a target to predict drugs with potential effects on ccRCC. In the high group, A.770041, AP. 24534, AUY922, AZ628 and AZD.0530 had lower IC50 values than the low group. Duan et al*.* found that A-770041 enhances the efficacy of paclitaxel and doxorubicin on tumor cells in osteosarcoma [36]. AP.24534 is also known as ponatinib. The current study shows that ponatinib is still controversial; the drug shows deep and durable responses in drug-resistant leukemia patients but has poor cardiovascular performance [37, 38]. In non-small cell lung cancer, AUY922 was resistant to cell therapy with TGF-β and paclitaxel-resistant phenotypes [39]. The AZ628 and BP-1-102 combination may have a positive therapeutic effect on KRAS lung cancer cells [40]. AZD.0530 is also known as saracatinib. In recent years, there have been many reports that saracatinib has been considered a potential therapeutic drug in various tumor treatments. In glioblastoma, the sensitivity of GBM cells and GSCs to radiotherapy can be enhanced by AZD0530 [41]. Saracatinib also has potential in the treatment of prostate and breast cancer, according to earlier reports. However, whether it has a positive effect on advanced patients is still being explored [42]. The results showed that five drugs may act as a vital part in ccRCC or improve the poor prognosis. Overall, LGALS3BP may be obviously correlated with the diagnosis and treatment of ccRCC, but the specific role and mechanism need to be further studied. Our study still has some limitations. First, the bioinformatics results of this article are all from an online database, and our results need more experimental or clinical data to be supported. Second, we did not continue to explore the related pathways of LGALS3BP in renal cancer due to time issues.

## CONCLUSIONS

In conclusion, our study demonstrates the relationship between LGALS3BP and proliferation, stemness and angiogenesis in clear cell renal cell carcinoma and indicates that LGALS3BP may be a potential prognostic biomarker. These results proved that LGALS3BP significantly promotes the proliferation of ccRCC cells. Five small-molecule drugs were predicted as potential therapeutics. These findings may provide new inspiration for clinical treatment ideas.

## MATERIALS AND METHODS

### Date and data processing

ccRCC data were obtained from TCGA, including 611 samples. All data use FPKM type.

### Analyses of LGALS3BP gene expression

TIMER 2.0 (http://timer.comp-genomics.org/) was used to analyze the expression changes in LGALS3BP between tumors and adjacent normal tissues. LGALS3BP mRNA levels in ccRCC patients were analyzed using UALCAN (http://ualcan.path.uab.edu/). The Human Protein Atlas (HPA) database (https://www.proteinatlas.org) was used to analyze the expression of LGALS3BP in clinical specimens.

### Prognostic characteristics of LGALS3BP

GEPIA2 was used to analyze the survival characteristics of LGALS3BP to obtain disease-free survival (DFS) and overall survival (OS) data. High and low expression groups were grouped by a cutoff value of 50% in GEPIA2.

### Nomogram prognostic model construction

We downloaded the ccRCC transcriptome and clinical data from the TCGA-KIRC database (https://portal.gdc.cancer.gov/). Independent prognostic factors in univariate and multivariate analyses using Cox logistic regression models had a significant impact on the prognosis of ccRCC. According to the results of the multivariate Cox regression analysis, we combined independent prognostic factors and the expression of LGALS3BP to draw a nomogram survival prediction map, which made the prediction of the 1-year, 3-year and 5-year survival rates of KIRC patients more reliable.

### Cell infiltration in the tumor microenvironment

TCGA tumor immune infiltration was analyzed using TIMER (https://cistrome.shinyapps.io/timer/) [43]. The Wilcoxon test was used to analyze the difference in LGALS3BP gene expression between the tumor and normal tissues. The relationship between LGALS3BP and immune cell infiltration was determined by a gene “module”. Immune cells were further analyzed using the “CIBERSORT” package. Correlation analysis was performed using the “vioplot” and “ggExtrat” packages.

The “estimate” package was used to score the tumor microenvironment. The “ggpubr”, “reshape2” and “limma” packages were used for visualization. We used the R software packages “ggplot2”, “reshape2”, “limma”, “corrplot” and “ggpubr” for immune checkpoint analysis, checked genes related to immune checkpoints, extracted the expression values of these genes, and observed the expression of immune checkpoints in ccRCC.

### LGALS3BP functional and disease analysis in ccRCC

CancerSEA (http://biocc.hrbmu.edu.cn/CancerSEA/) is the first database dedicated to decoding the different functional states of cancer cells at the single-cell level [44]. The CancerSEA database was used to analyze the functional status of LGALS3BP and explore the correlation and potential mechanism of LGALS3BP expression in ccRCC. Open Targets (https://platform.opentargets.org/) were used to analyze LGALS3BP-related diseases [45].

### Calculation of the stemness index (mRNAsi)

RNAseq data and corresponding clinical information for 530 ccRCC patients were obtained from The Cancer Genome Atlas (TCGA) dataset (https://portal.gdc.cancer.gov/). Using the OCLR algorithm to calculate mRNAsi, the mRNA expression-based signature contained a gene expression profile containing 11,774 genes. We used the same Spearman correlation coefficient (RNA expression data) and then used a linear transformation that subtracted the minimum value and divided it by the maximum value. The stemness index is mapped to the range [0,1].

### Gene screen associated with three phenotypes in kidney cancer

We screened 188 stemness-related genes in the relevant literature [46]. GeneCards was used to screen 100 genes with a strong correlation with proliferation and 50 genes with a strong correlation with angiogenesis (Supplementary Table 2). The pheatmap package was used to analyze the expression of the three phenotype-related genes. Venn diagrams were used to identify the three common phenotypic genes. The correlation between FGF2 and the phenotype was determined using Spearman correlation analysis. Sankey diagrams were used to analyze the impact of LGALS3BP, FGF2, and metastasis on patient prognosis.

### Cell culture and cell transfection

Human ccRCC (769-P and 786-O) and immortalized proximal tubule epithelial (HK2) cell lines were purchased from the Cell Bank of the Chinese Academy of Sciences.

1,640 medium (KeyGEN Biotech, Inc., Nanjing, China) was used to culture ccRCC cell lines. DMEM medium (KeyGEN Biotech, Inc.) was used to culture HK2 cell line. The culture environment was 5% carbon dioxide, and the serum concentration was 10% at 37° C LGALS3BP-siRNA and negative control siRNA were designed by Gima Genomics. 769-P cells were inoculated on 6-well plates and transfected according to the instructions of Lipofectamine 3000 (Invitrogen, Carlsbad, CA, USA) when the degree of fusion reached 50%. After culturing the cells for 48 h, functional experiments were performed. The sequence targeting LGALS3BP was 5’-GCGUGAACGAUGGUG ACAU-3’.

### Real-time polymerase chain reaction (RT–qPCR)

qRT**-**PCR analysis verified the LGALS3BP expression levels. Total RNA was extracted by TRIzol (TaKaRa Bio Inc. Shiga, Japan). qRT-PCR was performed on 7,500 FAST Real-Time PCR Systems (Applied Biosystems; Thermo Fisher Scientific, Waltham, MA, USA). Based on β-actin expression, the 2 ^– ΔΔ CT^ method was used to measure the relative expression of prognostic genes. The primers were as follows: LGALS3BP: Forward, CAGGAACCCAAGGCGTGAAC; Reverse, GTCAGGTCCCACAGGTTGTC; GAPDH: Forward, TGACTTCAACAGCGACACCCA; Reverse, CACCC-TGTTGCTGTAGCCAAA. LGALS3BP protein expression in clinical specimens was analyzed using the HPA database.

### Screening potential drugs for ccRCC

We divided LGALS3BP into high and low groups according to the median and used the “pRRophetic” package to analyze potential therapeutic drugs that may be related to ccRCC. The results are presented in a box diagram. Statistical significance was set at P < 0.05. Next, we used a public database (https://pubchem.ncbi.nlm.nih.gov/) to download the 3D conformer to screen for potential drugs.

### Cell counting kit-8 assay

After 24 h of transfection, 100 μl of 769-P cell and 786-O cell suspension at a density of 3x10^4^ cells/mL was added to each well of a 96-well plate, and 3 wells were used. After the cells returned to the normal state, 100 μl complete medium containing 10% Cell Counting Kit-8 (CCK-8) reagent was added to each well and incubated in darkness at 37° C for 2 h. The above operation was repeated at 0, 24, 48 and 72 h, and the absorbance was measured at 450 nm with a microplate reader (BioTek Instruments, Winooski, VT, USA) to detect cell proliferation.

### Statistical analyses

GraphPad Prism 9 software was used to analyze the experimental data and statistical charting. Independent samples t tests were used for comparisons between two samples and paired samples were used for paired t tests. Pearson’s X2 test was used to estimate the correlation between LGALS3BP expression and clinical features. The immune checkpoint with a significant difference between the two groups was compared using the Wilcoxon test. Cox regression analysis was used to analyze univariate and multivariate factors affecting patient prognosis. Statistical significance was determined as follows: *p < 0.05, **p < 0.01, and ***p < 0.001.

### Availability of data and materials

The original contributions presented in the study are included in the article/Supplementary Material, further inquiries can be directed to the corresponding author.

## Supplementary Material

Supplementary Figure 1

Supplementary Table 1

Supplementary Table 2
