# Supplementary figures and images for: LGALS3BP is a novel and potential biomarker in clear cell renal cell carcinoma
Source: Aging (Albany NY). 2024 Feb 22;16(4):4033–51. doi: 10.18632/aging.205578 (PMC10929836; doi:10.18632/aging.205578)

SUPPLEMENTARY FIGURE

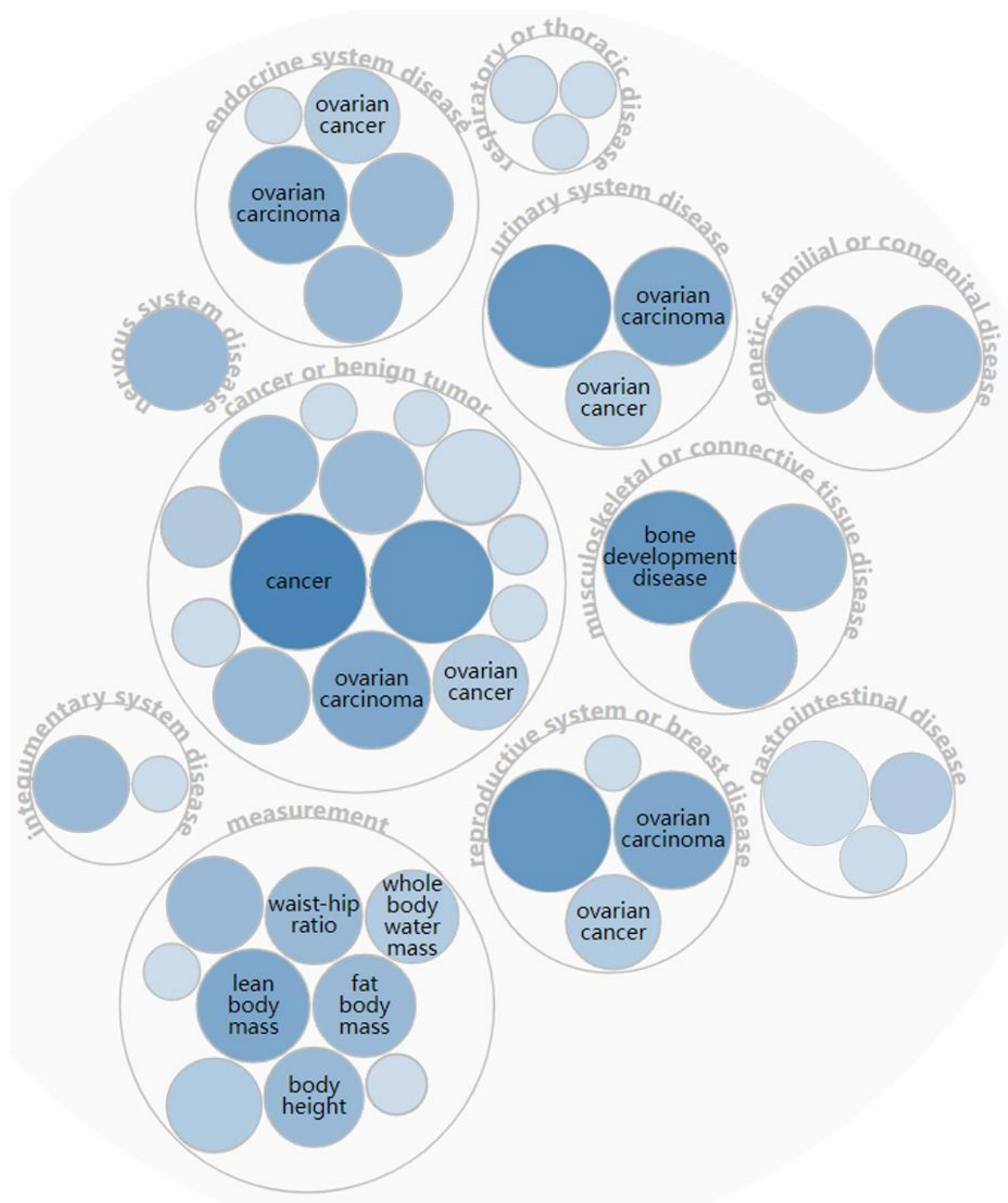

Supplementary Figure 1. FGF2 has been implicated in cancer.

Supplement: Supplementary Figure 1 [file aging-16-205578-s001.pdf]
