# Supplementary material for: LGALS3BP is a novel and potential biomarker in clear cell renal cell carcinoma
Source: Aging (Albany NY). 2024 Feb 22;16(4):4033–51. doi: 10.18632/aging.205578 (PMC10929836; doi:10.18632/aging.205578)
Supplement: Supplementary Table 1 [file aging-16-205578-s002.pdf]

SUPPLEMENTARY TABLE

Supplementary Table 1. Differential expression of LGALS3BP in pancancer.

Tumor	Nor	p
BLCA.Tumor (n=408)	BLCA.Normal (n=19)	0.0000297497946015658
BRCA.Tumor (n=1093)	BRCA.Normal (n=112)	4.30384106899536E-09
CESC.Tumor (n=304)	CESC.Normal (n=3)	0.122156585103806
CHOL.Tumor (n=36)	CHOL.Normal (n=9)	6.7707623608152E-08
COAD.Tumor (n=457)	COAD.Normal (n=41)	0.0378385206901153
ESCA.Tumor (n=184)	ESCA.Normal (n=11)	0.0000159822113358476
GBM.Tumor (n=153)	GBM.Normal (n=5)	0.00848515671774974
HNSC-HPV+.Tumor (n=97)	HNSC-HPV-.Tumor (n=421)	1.21518071910518E-08
HNSC.Tumor (n=520)	HNSC.Normal (n=44)	1.16798977768297E-16
KICH.Tumor (n=66)	KICH.Normal (n=25)	0.0000457064256399929
KIRC.Tumor (n=533)	KIRC.Normal (n=72)	0.00363559852729953
KIRP.Tumor (n=290)	KIRP.Normal (n=32)	1.72163242771148E-11
LIHC.Tumor (n=371)	LIHC.Normal (n=50)	0.037692508018037
LUAD.Tumor (n=515)	LUAD.Normal (n=59)	2.68098936543446E-16
LUSC.Tumor (n=501)	LUSC.Normal (n=51)	0.64760328430446
PAAD.Tumor (n=178)	PAAD.Normal (n=4)	0.0414187390759963
PCPG.Tumor (n=179)	PCPG.Normal (n=3)	0.144660752037478
PRAD.Tumor (n=497)	PRAD.Normal (n=52)	1.54661961829195E-14
READ.Tumor (n=166)	READ.Normal (n=10)	0.675514110492107
SKCM.Tumor (n=103)	SKCM.Metastasis (n=368)	0.456846317195677
STAD.Tumor (n=415)	STAD.Normal (n=35)	0.0000036775318112862
THCA.Tumor (n=501)	THCA.Normal (n=59)	0.0055579786665661
UCEC.Tumor (n=545)	UCEC.Normal (n=35)	1.56351361465094E-08
